# COPII proteins exhibit distinct subdomains within each ER exit site for executing their functions

**DOI:** 10.1038/s41598-019-43813-3

**Published:** 2019-05-14

**Authors:** Miharu Maeda, Kazuo Kurokawa, Toshiaki Katada, Akihiko Nakano, Kota Saito

**Affiliations:** 1 Department of Biological Informatics and Experimental Therapeutics, Graduate School of Medicine, Akita University 1-1-1, Hondo, Akita 010-8543 Japan; 2 Live Cell Super-Resolution Imaging Research Team, RIKEN Center for Advanced Photonics, 2-1 Hirosawa, Wako, Saitama 351-0198 Japan; 3 Faculty of Pharmacy, Musashino University, Tokyo, 202-8585 Japan

**Keywords:** Coat complexes, Endoplasmic reticulum, Secretion

## Abstract

Secretory proteins are exported from special domains of the endoplasmic reticulum (ER) termed ER exit sites, via COPII-coated carriers. We recently showed that TANGO1 and Sec16 cooperatively organize mammalian ER exit sites for efficient secretion. However, the detailed spatial organization of mammalian ER exit sites is yet to be revealed. Here, we used super-resolution confocal live imaging microscopy (SCLIM) to investigate the localization of endogenous proteins, and we identified domains abundant in transmembrane complexes (TANGO1/cTAGE5/Sec12) juxtaposed to Sec16. Interestingly, this domain can be distinguished from the inner and the outer coats of COPII proteins within each mammalian ER exit site. Cargoes are partially concentrated in the domain for secretion. Our results suggest that mammalian ER exit sites compartmentalize proteins according to their function in COPII vesicle formation.

## Introduction

Secretory proteins are exported from specialized domains of the endoplasmic reticulum (ER) termed ER exit sites, via COPII-coated carriers^1^. The formation of COPII-coated carriers is initiated upon activation of small GTPase Sar1 by its guanine-nucleotide exchange factor, Sec12. Activated Sar1 exposes an N-terminal amphipathic helix, which is inserted into ER membranes, and induces membrane curvature required for vesicle budding. Sar1 then recruits the COPII-inner coat complex, Sec23/Sec24. Sec24 binds to cargoes, whereas Sec23 acts as a GTPase-activating protein for Sar1. The outer coat complex, Sec13/Sec31 then interacts with Sec23/Sec24, and this interaction enhances the GTPase-activating protein activity of Sec23, and completes coat assembly. Sec16 is another peripheral protein that is involved in COPII coat assembly at ER exit sites by interacting with multiple COPII proteins. Although key proteins for COPII-vesicle formation are well conserved among species, in mammals, several additional proteins are vital for maintaining correct and functional ER exit site structures^2^. We recently showed that mammalian ER exit sites comprise two macromolecular complexes, TANGO1L/cTAGE5/Sec12 (900 kDa) and TANGO1S/cTAGE5/Sec12 (700 kDa)^3^. TANGO1L was originally identified as a collagen cargo receptor^4^, but we recently found that both TANGO1L and TANGO1S are required not only for collagen secretion, but also for maintaining proper ER exit site organization by directly interacting with Sec16^5^. These results indicated that mammalian ER exit sites have distinct characteristics compared to the counterparts in other organisms, such as yeast. Electron microscopic analysis suggests that mammalian ER exit sites are approximately 400 nm in size, and stable ER exit sites consist of 2 to 6 COPII-coated structures on average^6,7^. However, electron microscopy failed to show the spatial organization of proteins constituting the ER exit sites. Here, we used super-resolution confocal live imaging microscopy (SCLIM) to investigate the localization of endogenous proteins at ER exit sites, and we identified domains abundant in transmembrane complexes (TANGO1/cTAGE5/Sec12) juxtaposed to Sec16. Interestingly, this domain could be distinguished from the inner and the outer coats of COPII proteins. Moreover, cargoes are partially concentrated into the domain for secretion. Our results suggest that mammalian ER exit sites compartmentalize proteins according to their function for COPII-vesicle formation.

## Results and Discussion

### Each ER exit site is composed of subdomains

**Figure 1:**
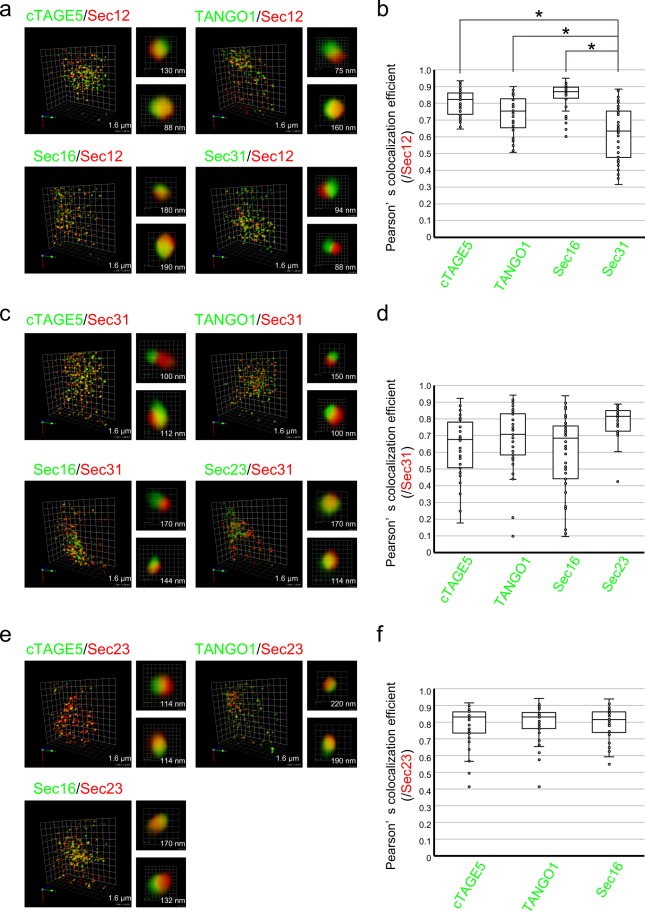
COPII components show distinct localization within each ER exit site. (**a**) HeLa cells were fixed and costained with anti-cTAGE5 (green) or anti-TANGO1 (green) or anti-Sec16 (green) or anti-Sec31 (green) and anti-Sec12 (red) antibodies. 3D dual-color observation by SCLIM is shown. Right, magnifications of images on the left with two-dimensional (2D) projection. The length indicates the scale of each unit. (**b**) Quantification of Pearson’s correlation coefficient to quantify the degree of colocalization in (**a**). n = 40 (eight ER exit sites in 5 cells). (**c**) HeLa cells were fixed and costained with anti-cTAGE5 (green) or anti-TANGO1 (green) or anti-Sec16 (green) or anti-Sec23 (green) and anti-Sec31 (red) antibodies. 3D dual-color observation by SCLIM is shown. Right, magnifications of images on the left with 2D projection. The length indicates the scale of each unit. (**d**) Quantification of Pearson’s correlation coefficient to quantify the degree of colocalization in (**c**). n = 40 (eight ER exit sites in 5 cells). (**e**) HeLa cells were fixed and co-stained with anti-cTAGE5 (green) or anti-TANGO1 (green) or anti-Sec16 (green) and anti-Sec23 (red) antibodies. 3D dual color observation by SCLIM is shown. Right, magnifications of images on the left with 2D projection. The length indicates the scale of each unit. (**f**) Quantification of Pearson’s correlation coefficient to quantify the degree of colocalization in. (**e**) n = 40 (eight ER exit sites in 5 cells). Results in (**b**,**d**,**f**) are displayed as box-and-whisker plots (whiskers represent 1.5× interquartile range) *P < 0.05.

A significant amount of knowledge has been accumulated on the interaction properties of proteins localized at ER exit sites. However, it has not been fully investigated how these proteins spatially organize the structure of ER exit sites. In our previous studies, we developed antibodies against COPII components, and most of them could be used to visualize endogenous proteins, including cTAGE5 (rabbit polyclonal), TANGO1 (rabbit polyclonal), Sec16 (rabbit polyclonal), Sec12 (rat monoclonal) and Sec23 (rat monoclonal)^4,5,8,9^. Utilizing these antibodies, we checked the localization of endogenous proteins of ER exit sites by three-dimensional (3D) dual-color high-resolution image analysis by super-resolution confocal live imaging microscopy (SCLIM). Cold methanol-fixed HeLa cells were double-stained with primary antibodies against COPII proteins followed by Alexa Fluor-conjugated secondary antibodies. Before acquiring images, we calibrated and adjusted color registration of the microscope with fluorescent beads. First, we evaluated the colocalization rate of several COPII proteins visualized with Alexa Fluor 488 and Sec12 visualized with Alexa Fluor 568 (Fig. 1a). We measured the colocalization efficiency of forty ER exit sites in five cells (Fig. 1b). The colocalization efficiencies of Sec12 and cTAGE5, Sec12 and TANGO1, and Sec12 and Sec16, fell into a similar range. Conversely, the colocalization efficiency of Sec12 and Sec31 was significantly lower and more variable (Fig. 1b). These results suggested that each ER exit site has a similar organization of Sec12 and cTAGE5, Sec12 and TANGO1, and Sec12 and Sec16, whereas the organization of Sec12 and Sec31 seems to vary among ER exit sites. The results were confirmed in dye-exchange experiments (Fig. S1a,b).

Next, we investigated the colocalization rate of Sec31 with various COPII proteins. As shown in Fig. 1c,d, the colocalization rates of Sec31 and cTAGE5, Sec31 and TANGO1, Sec31 and Sec16 varied, suggesting that each ER exit site varies in terms of spatial organization of Sec31 and other COPII components. Conversely, the colocalization rate of Sec31 and Sec23 was rather constant, indicating that Sec23 and Sec31 maintain a constant spatial organization among ER exit sites. The results were confirmed in dye-exchange experiments (Fig. S1c,d). Next, we measured the colocalization of COPII proteins with Sec23. cTAGE5, TANGO1, and Sec16 colocalized extensively with Sec23 (Figs 1e,f and S1e,f). These data suggested that proteins at ER exit sites are not uniformly localized, but form domains within the ER exit sites. Finally, we sorted the data to show the colocalization efficiencies of cTAGE5, TANGO1, and Sec16 (Fig. S2). The rearranged data clearly showed that both Sec12 and Sec23 colocalize better with cTAGE5, TANGO1, and Sec16 than with Sec31, although statistical significance was not reached for all comparisons (Fig. S2). These data, together with the interaction properties of proteins localized at ER exit sites so far, showed that proteins known to interact with each other yield higher colocalization efficiencies compared to the proteins without interactions.

### Cargoes are concentrated into subdomains for secretion

**Figure 2:**
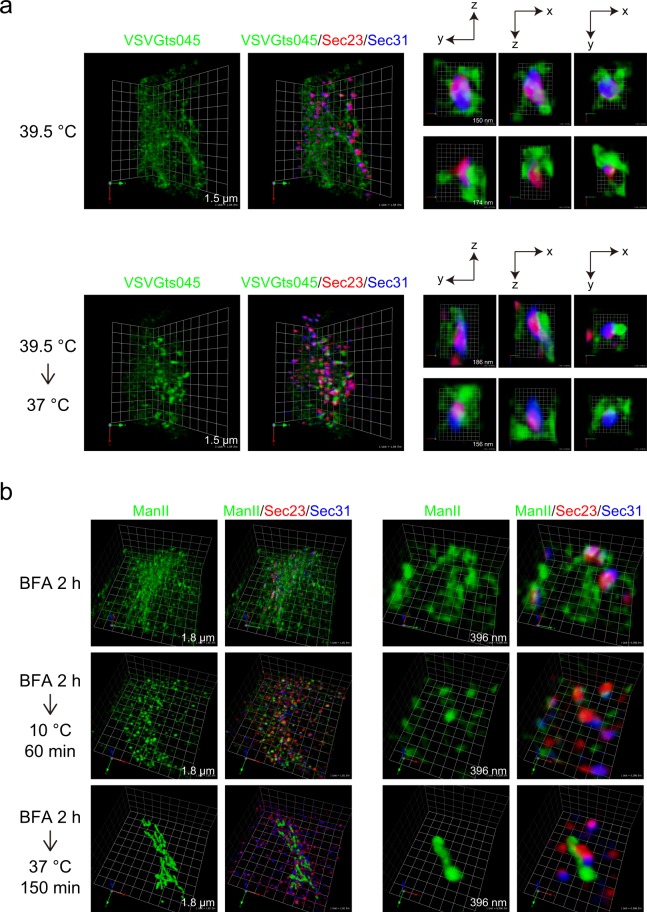
Cargoes are concentrated into domains within the ER exit sites for secretion. (**a**) HeLa cells were transfected with VSVG-ts045-GFP. The cells were cultured at 39.5 °C to accumulate the protein within the ER, and fixed (upper panel), or further incubated at 37 °C for 8 min before fixation (bottom panel). The cells were stained with anti-Sec23 (red) and anti-Sec31 (blue) antibodies. 3D triple-color observation by SCLIM is shown. Right, magnifications of images on the left with 2D projection. The length indicates the scale of each unit. (**b**) HeLa cells stably expressing ManII-GFP were incubated with 5 µg/ml brefeldinA for 2 h, washed with ice-cold DMEM supplemented with 10% fetal bovine serum, and incubated either at 10 °C for 60 min or at 37 °C for 150 min before fixation. Fixed cells were processed for immunofluorescence. The cells were stained with anti-Sec23 (red) and anti-Sec31 (blue) antibodies. 3D triple-color observation by SCLIM is shown. Right, magnifications of images on the left. The length indicates the scale of each unit.

**Figure 3:**
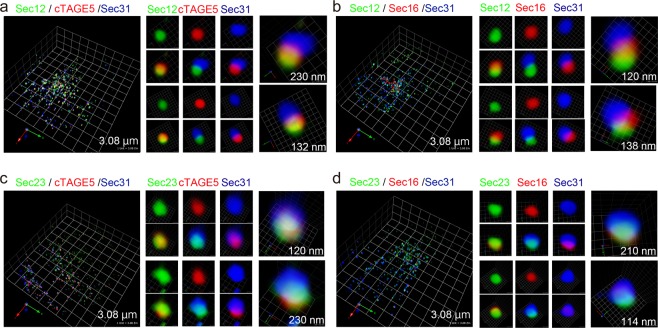
Sec31 is distant from most of the COPII components at ER exit sites. HeLa cells were fixed and costained with anti-Sec12 (green), anti-cTAGE5 (red) and anti-Sec31 (blue) (**a**); anti-Sec12 (green), anti-Sec16 (red) and anti-Sec31 (blue) (**b**); anti-Sec23 (green), anti-cTAGE5 (red) and anti-Sec31 (blue) (**c**); anti-Sec23 (green), anti-Sec16 (red) and anti-Sec31 (blue) (**d**) antibodies. 3D triple -color observation by SCLIM is shown. Right, magnifications of images on the left with 2D triple-color projection. The length indicates the scale of each unit.

We examined VSVG-ts045-GFP trafficking in HeLa cells to know the positions of ER exit sites relative to the ER membranes and cargoes. When cells expressing VSVG-ts045-GFP were incubated at 39.5 °C, VSVG-ts045-GFP was diffused within the ER structure, as previously reported (Fig. 2a, upper panel)^10^. 3D triple-color observation of Sec23 and Sec31 revealed that ER exit sites were surrounded with ER membranes, consistent with previous electron-microscopic findings (Fig. 2a, upper panel, right)^6^. Staining of Sec23 and Sec31 showed that these two proteins were not completely merged, but significantly overlapped. However, in this condition, we could not resolve the spatial arrangement of Sec23, Sec31, and ER structures. We further incubated the cells at 37 °C for 8 min, allowing VSVG-ts045-GFP to be concentrated at ER exit sites, as previously shown (Fig. 2a, bottom panel)^5^. Some of the VSVG signals were still observed as reticular patterning, but most were concentrated and formed dot-like structures, some of which significantly overlapped with Sec23 and Sec31 signals. 3D triple-color microscopy indicated that Sec23 signals were consistently located between VSVG and Sec31 signals, suggesting that the organization of ER exit sites preserves the spatial relation of the inner and outer coat complexes of COPII-coat structures (Fig. 2a, bottom panel, right). We verified the above results by analyzing Mannosidase II (ManII) transport from ER to Golgi. HeLa cells stably expressing ManII-GFP were treated with brefeldin A to redistribute ManII from Golgi to ER. As shown in Fig. 2b, top panel, cargoes were not merged with Sec23 and Sec31 in this condition. After removal and washout of brefeldin A, the cells were incubated at 10 °C to accumulate ManII to the ER exit site^11^. Consequently, ManII-GFP was concentrated into the structure that was significantly overlapped with Sec23 and Sec31 signals, suggesting that ManII-GFP was concentrated at the ER exit sites (Fig. 2b, middle panel). When the cells were incubated at 37 °C after washout, cargoes were transported to the Golgi (Fig. 2b, bottom panel). To investigate the spatial organization of proteins at ER exit sites further, we conducted 3D triple-color analysis of endogenous proteins by SCLIM. Consistent with dual-color observation, cTAGE5/Sec12, Sec12/Sec16 extensively colocalized, whereas Sec31 showed variable colocalization with cTAGE5, Sec12, and Sec16 (Fig. 3a,b; Videos S1 and S2). In contrast, Sec23 signals were relatively close to those of all proteins evaluated, including Sec16, cTAGE5, and Sec31 (Fig. 3c,d; Videos S3 and S4). These data further supported the idea that the structures of ER exit sites are maintained based on the protein interaction property of each constituent.

In this study, 3D triple-color high-resolution observation by SCLIM clearly showed that each ER exit site is composed of subdomains, and domain organization is based on the interaction property of the proteins constituting the ER exit sites. TANGO1/cTAGE5/Sec12 is a complex located within the ER membranes, and our former findings^5^ and current colocalization data suggest that Sec16 is closely associated with this complex. By performing experiments utilizing VSVG and ManII-GFP, we showed that Sec23 is located between Sec31 and the ER membrane.

**Figure 4:**
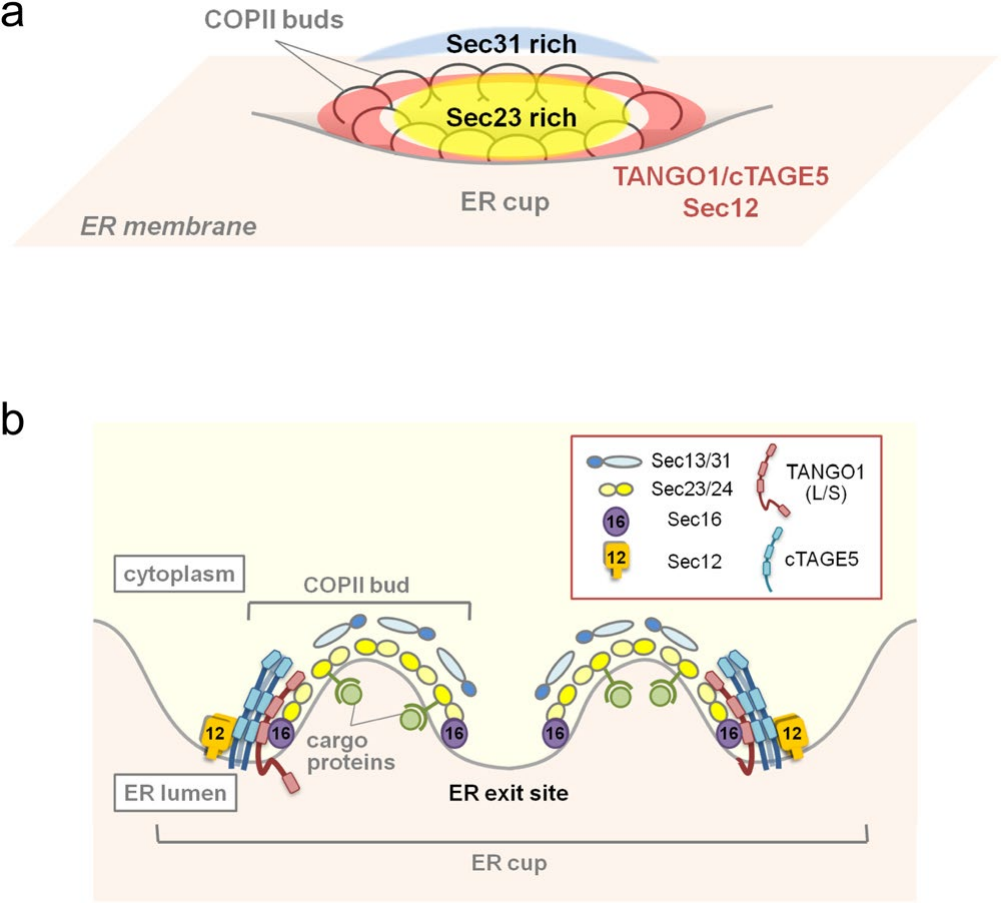
Model for mammalian ER exit site organization. (**a**) Schematic representation of ER exit site organization. (**b**) Cross-sectional view of the ER exit site presented in (**a**).

Based on our observations and former electron microscopic analysis^6^, we propose a model for ER exit site organization as shown in Fig. 4a. TANGO1/cTAGE5/Sec12 complex forms the scaffold of the ER exit sites probably with symmetrical positioning. Sec16 would closely interact with the complex within the ER membrane. Sec23/Sec24 complex then interacts with the scaffold complex at the cytoplasmic face, followed by interaction with the outer coat complex Sec13/Sec31 (Fig. 4b). To our knowledge, very few studies have investigated the localization of endogenous proteins in detail by super-resolution microscopy. As reported before, SCLIM works best on live cells^12^. Thus, this study provided an important basis for live imaging analysis of ER protein structures in future. Moreover, a new generation of SCLIM instruments with even higher temporal and spatial resolution is forthcoming. We plan to study how the organization of ER exit sites as reported here reacts to cargo in the near future.

## Methods

### Antibodies

Anti-cTAGE5 (rabbit), TANGO1 (rabbit), Sec16 (rabbit), Sec12 (rat) and Sec23 (rat) antibodies were made as described previously^5,8,9^. Mouse anti-Sec31 antibody was purchased from BD Biosciences.

### Cell culture and transfection

HeLa cells were cultured in DMEM supplemented with 10% foetal bovine serum. For plasmids transfection, Fugene 6 (Promega) was used according to the manufacturer’s protocol. Doxycycline-inducible stable HeLa cell lines expressing ManII-GFP were made with a lentivirus system described previously^3,13^.

### VSVG-transport assay

HeLa cells were transfected with VSVG-ts045-GFP and further incubated for 8 h at 37 °C. Then, the cells were shifted to 39.5 °C for 12 h to retain the protein in the ER. Cells were replaced with ice-cold medium and placed on ice for 1 min. Then, the cells were incubated for indicated times before fixation. Fixed cells were processed for immunofluorescence.

### ManII-GFP transport assay

ManII-GFP was induced with 100 ng/ml doxycycline for 24 h in ManII-GFP stable cell lines. The cells were then incubated with 5 µg/ml brefeldinA for 2 h, washed with ice-cold DMEM supplemented with 10% fetal bovine serum and incubated either at 10 °C for 60 min or at 37 °C for 150 min before fixation. Fixed cells were processed for immunofluorescence.

### Immunofluorescence Microscopy

HeLa cells grown on coverslips were washed with PBS, fixed with methanol (6 min at −20 °C), and then washed with PBS and blocked in blocking solution (5% BSA in PBS with 0.1% Triton X-100 for 30 min). After blocking, cells were stained with primary antibody (1 h at room temperature) followed by incubation with Alexa Fluor-conjugated secondary antibodies (Alexa Fluor 488, Alexa Fluor 568, and/or Alexa Fluor 688 for 1 h at room temperature). SCLIM analysis was essentially performed as described previously^12^. SCLIM was achieved with a Olympus model IX-71 inverted fluorescence microscope with a UPlanSApo 100× NA 1.4 oil objective lens (Olympus, Japan), a high-speed spinning-disk confocal scanner (Yokogawa Electric, Japan), a custom-made spectroscopic unit, image intensifiers (Hamamatsu Photonics, Japan) with a custom-made cooling system, and two EM-CCD cameras (Hamamatsu Photonics, Japan) for green and red observation. To increase the spatial resolution, a magnification lens (4× or 10×) was put in the light path between the confocal scanner and the spectroscopic unit (final magnification, ×267 or ×667). For 3D images, we collected optical sections spaced 100 nm apart by oscillating the objective lens vertically with a custom-made piezo actuator (Yokogawa Electric) that oscillated in the *z*-axis position at a high-repetition rate (100 µm at 10–30 Hz) and a fine step (minimum movement is 50 nm apart). 3D images were reconstructed and deconvolved through point-spread functions optimized for a spinning-disk confocal scanner using Volocity software (Perkin Elmer, MA).

## Supplementary information

Supplementary Information

Video S1

Video S2

Video S3

Video S4

## Data Availability

The data that support the findings of this study are available from the corresponding author upon request.
