# Supplementary material for: COPII proteins exhibit distinct subdomains within each ER exit site for executing their functions
Source: Sci Rep. 2019 May 14;9:7346. doi: 10.1038/s41598-019-43813-3 (PMC6517409; doi:10.1038/s41598-019-43813-3)
Supplement: Supplementary file 1 — Supplementary Information [file 41598_2019_43813_MOESM1_ESM.pdf]

# Supplementary Information

# COPII proteins exhibit distinct subdomains within each ER exit site for executing their functions

Miharu Maeda[1#,] Kazuo Kurokawa[2#*], Toshiaki Katada[3], Akihiko Nakano[2] and Kota Saito[1*]

[1]Department of Biological Informatics and Experimental Therapeutics, Graduate School of Medicine, Akita University 1-1-1, Hondo, Akita, 010-8543, Japan
[2]Live Cell Super-Resolution Imaging Research Team, RIKEN Center for Advanced Photonics, 2-1 Hirosawa, Wako, Saitama, 351-0198, Japan
[3]Faculty of Pharmacy, Musashino University, Tokyo, 202-8585, Japan

[#] These authors contributed equally to this work

[*] Correspondence and requests for materials should be address to:
K.K. (kkurokawa@riken.jp) and K.S. (ksaito@med.akita-u.ac.jp)

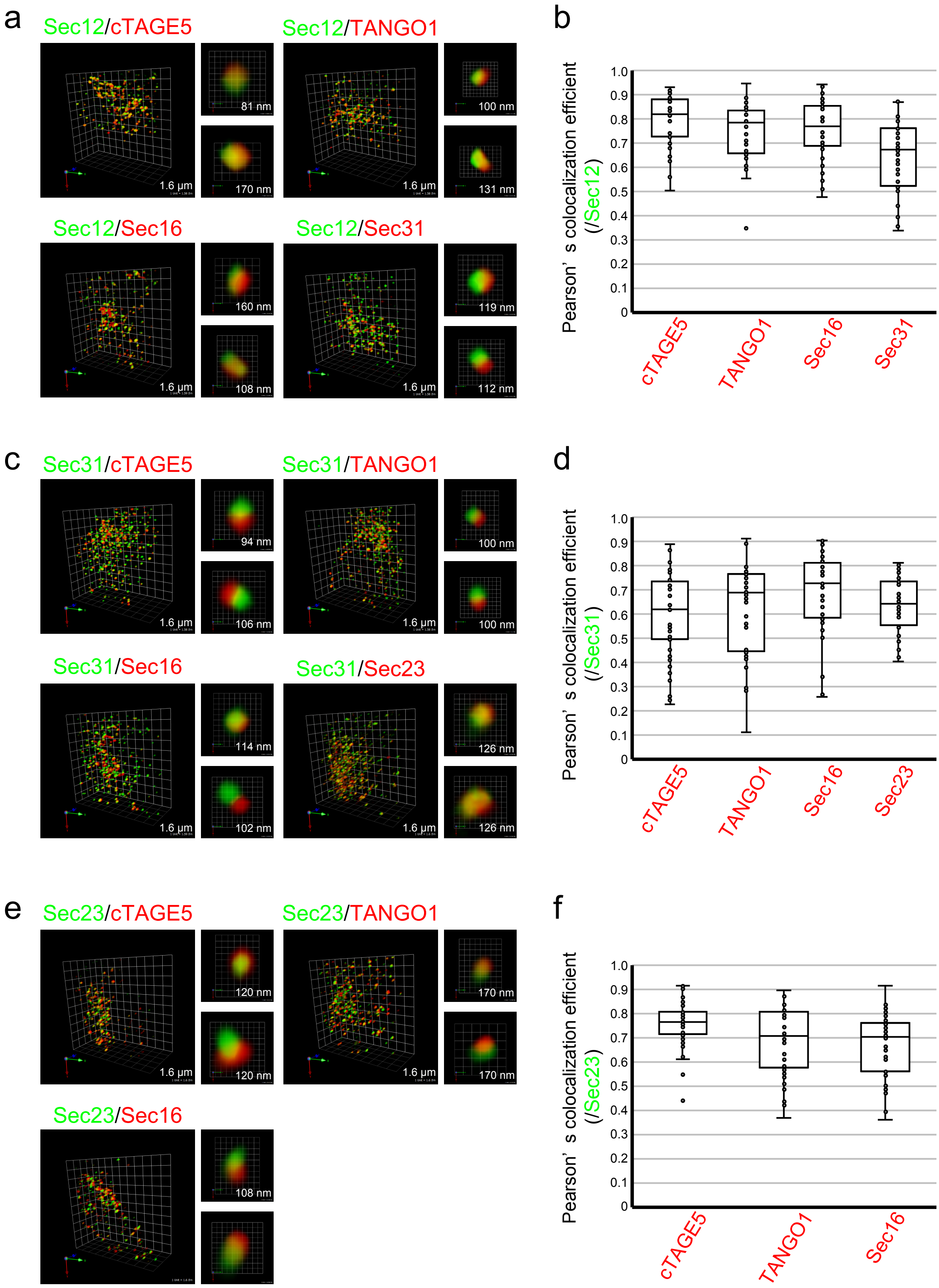


**Figure S1. Dye-exchanged experiments of Figure 1.** (a) HeLa cells were fixed and co-stained with anti-Sec12 (green) and anti-cTAGE5 (red) or anti-TANGO1 (red) or anti-Sec16 (red) or anti-Sec31 (red) antibodies. 3D dual-color observation by SCLIM is shown. Right, magnifications of images on the left with 2D projection. The length indicates the scale of each unit. (b) Quantification of Pearson’ s correlation coefficient to quantify the degree of colocalization in a. n = 40 (eight ER exit sites in 5 cells). (c) HeLa cells were fixed and costained with anti-Sec31 (green) and anti-cTAGE5 (red) or anti-TANGO1 (red) or anti-Sec16 (red) or anti-Sec23 (red) antibodies. 3D dual-color observation by SCLIM is shown. Right, magnifications of images on the left with 2D projection. The length indicates the scale of each unit. (d) Quantification of Pearson’ s correlation coefficient to quantify the degree of colocalization in c. n = 40 (eight ER exit sites in 5 cells). (e) HeLa cells were fixed and costained with anti-Sec23 (green) and anti-cTAGE5 (red) or anti-TANGO1 (red) or anti-Sec16 (red) antibodies. 3D dual-color observation by SCLIM is shown. Right, magnifications of images on the left with 2D projection. The length indicates the scale of each unit. (f) Quantification of Pearson’ s correlation coefficient to quantify the degree of colocalization in e. n = 40 (eight ER exit sites in 5 cells). Results in b, d and f are displayed as box-and-whisker plots (whiskers represent 1.5x interquartile range). *, $P < 0.05$.

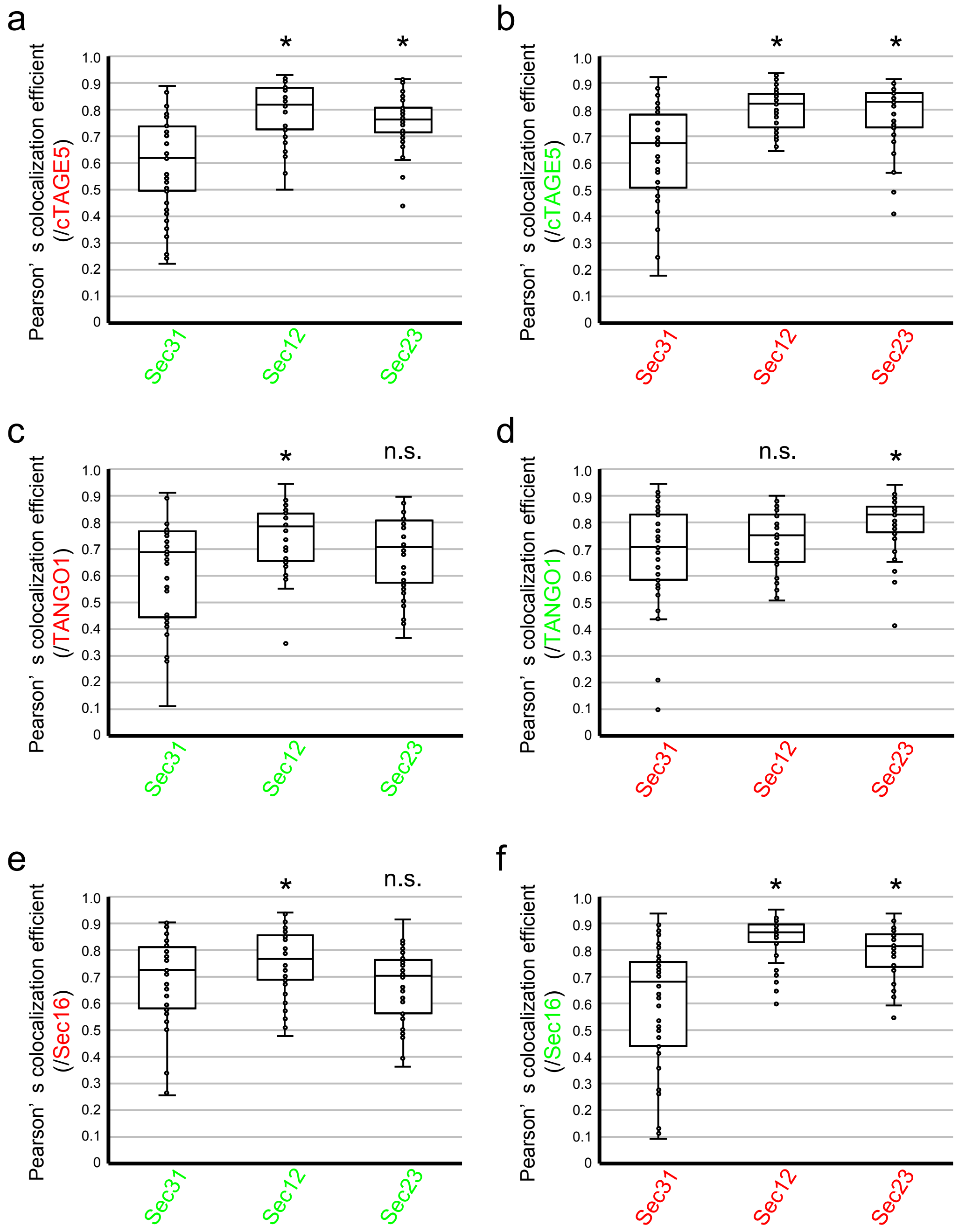


**Figure S2. Rearranged data from Figure 1 and S1.** Quantification of Pearson' s correlation coefficient to quantify the degree of colocalization. n = 40 (eight ER exit sites in 5 cells). Results are displayed as box-and-whisker plots (whiskers represent 1.5x interquartile range). *, $P < 0.05$ compared with Sec31. n.s., not significant compared with Sec31.

**Video S1. Magnified 3D image of Sec12 (green), cTAGE5 (red), and Sec31 (blue).** 3D multi-angle views of one selected ER exit sites in Figure 3A (upper right) is shown.

**Video S2. Magnified 3D image of Sec12 (green), Sec16 (red), and Sec31 (blue).** 3D multi-angle views of one selected ER exit sites in Figure 3B (upper right) is shown.

**Video S3. Magnified 3D image of Sec23 (green), cTAGE5 (red), and Sec31 (blue).** 3D multi-angle views of one selected ER exit sites in Figure 3C (upper right) is shown.

**Video S4. Magnified 3D image of Sec23 (green), Sec16 (red), and Sec31 (blue).** 3D multi-angle views of one selected ER exit sites in Figure 3D (upper right) is shown.
